# Drugs commonly associated with weight change: umbrella systematic review and meta-analysis (Protocol)

**DOI:** 10.1186/2046-4053-1-44

**Published:** 2012-09-29

**Authors:** Juan Pablo Domecq, Gabriela Prutsky, Zhen Wang, Tarig Elraiyah, Juan Pablo Brito, Karen Mauck, Mohammed H Lababidi, Aaron Leppin, Salman Fidahussein, Larry J Prokop, Victor M Montori, Mohammad H Murad

**Affiliations:** 1 Knowledge and Evaluation Research Unit, Mayo Clinic, Rochester, MN, USA; 2 Unidad de Conocimiento y Evidencia, Universidad Peruana Cayetano Heredia, Lima, Peru; 3 Division of Preventive, Occupational and Aerospace Medicine, Mayo Clinic, Rochester, MN, USA; 4 Division of Endocrinology, Diabetes, Metabolism, Nutrition, Mayo Clinic, Rochester, MN, USA; 5 Division of General Internal Medicine, Mayo Clinic, Rochester, MN, USA; 6 Mayo Clinic Libraries, Mayo Clinic, Rochester, MN, USA; 7 Mayo Clinic, The Knowledge and Evaluation Research Unit, 200 First Street SW, Rochester, MN, 55905, USA

**Keywords:** Drug, Adults, Obesogenic, Leptogenic, Weight loss, Weight gain, Weight change, Systematic review

## Abstract

**Background:**

Many drugs and treatments given to patients for various reasons affect their weight. This side effect is of great importance to patients and is also a concern for the treating physician because weight change may lead to the emergence or worsening of other health conditions.

**Objective:**

The aim of this study is to summarize the evidence about commonly prescribed drugs and their association with weight change.

**Methods/Design:**

Umbrella systematic review and meta-analysis of randomized controlled trials.

We will use an umbrella approach to identify eligible randomized controlled trials (RCTs). We will search for systematic reviews of RCTs that compare any of the drugs that have been associated with weight gain (obesogenic) or weight loss (leptogenic); these have been summarized by our experts’ panel in a predefined list. Two reviewers will independently determine RCT eligibility. Disagreement will be solved by consensus and arbitrated by a third reviewer. We will extract descriptive, methodological, and efficacy data in duplicate. Our primary continuous outcomes will be weight loss or gain expressed as a mean difference (MD) for weight (kg) or BMI (kg/m^2^). We will calculate the MD considering the mean difference in weight or BMI between baseline and the last available follow-up in both study arms (drugs and placebo). Our primary dichotomous outcome, presented as a relative risk, will compare the ratio of the incidence of weight change in each trial arm. When possible, results will be pooled using classic random-effects meta-analyses and a summary estimate with 95% confidence interval will provided. We will use the I^2^ statistic and Cochran’s Q test to assess heterogeneity. The risk of bias will be assessed using the Cochrane risk of bias tool. Publication bias, if appropriate, will be evaluated, as well as overall strength of the evidence.

**Discussion:**

This systematic review will offer the opportunity to generate a ranking of commonly prescribed drugs in terms of their effect on weight, allowing guideline developers and patient-physician dyad to choose between available therapies.

## Background

Many drugs and treatments given to patients for various reasons affect their weight. This effect is of great importance to patients and is also a concern for the treating physician because weight change may lead to the emergence or worsening of other health conditions. Currently, obesity is considered a worldwide epidemic and one of the most important public health problems, mainly in, but not restricted to, developed countries [1]. In the USA, the lifetime risk of developing obesity is 25% [2]. According to World Health Organization global estimates over 200 million men and nearly 300 million women were obese worldwide in 2008 [3]. Similarly, being underweight is associated with increased mortality from non-cancer and non-cardiovascular causes, including diabetes, kidney diseases, acute and chronic respiratory diseases, and others [4], and obesity is associated with an increased risk of death from any cause [5,6] as also from cardiovascular causes [4].

## Study objectives

The aim of this systematic review is to summarize the evidence about commonly prescribed drugs and their association with weight loss or weight gain. In each of the included studies we will assess the risk of bias, the overall quality of evidence, and the magnitude (continuous outcome) and incidence (dichotomous outcome) of weight change. This evidence will help guideline developers, clinicians, and patients in: (1) choosing the most appropriate therapy for their condition based on their weight goal; and (2) anticipate and manage the weight change associated with therapy.

Thus, individualized treatment approaches that include therapy choice and monitoring strategies can be recommended based on the overall clinical context, patient’s values, and preferences.

## Methods/Design

Due to the large number of drugs and the availability of multiple systematic reviews of these drugs, we decided to conduct an ‘umbrella’ search strategy to identify eligible randomized controlled trials (RCTs). We will identify first systematic reviews (SR) that compare our drug of interest to placebo. Eligible SR will be used to identify relevant RCTs. For the drugs that we are unable to identify existing SR, we will conduct new searches to identify RCTs.

### Choosing drugs for the systematic review

There are several drugs that have been associated with weight gain (obesogenic) or weight loss (leptogenic). Members from the Endocrine Society (ES), experts in the field, developed for each group a list of commonly prescribed drug families and specific drugs (Table 1). These lists include, in the leptogenic group (List A) six drug classes and 26 specific drugs, and in the obesogenic group (List B) five drug classes and 27 specific drugs. Noteworthy, there is a possibility that, by the end of this systematic review, some of the listed drugs will be classified as weight neutral or even in a different group than were classified by the experts form the ES in the previous mentioned list.

**Table 1:** Intervention lists

**List A (Leptogenic)**		**List B (Obesogenic)**	
*Drug classes*		*Drug classes*	
1.	ACE inhibitors	1.	Insulin I.M or S.C.
2.	Calcium channel blockers	2.	Oral nasal decongestants
3.	MAOIs	3.	Protease inhibitors
4.	NSAIDs	4.	Sulfonylureas
5.	Oral antihistamines	5.	Oral hormonal contraceptives containing progestational steroids
6.	Oral corticosteroids		
*Specific drugs*^a^		*Specific drugs*^a^	
1.	Acarbose	1.	Amitriptyline
2.	Aripiprazole	2.	Atenolol
3.	Bromocriptine	3.	Carbamazepine
4.	Bupropion	4.	Citalopram
5.	Bupropion + Naltrexone	5.	Clozapine
6.	Cabergoline	6.	Doxazosin mesylate
7.	Duloxetine	7.	Doxepin
8.	Exenatide	8.	Escitalopram
9.	Fluoxetine^b^	9.	Fluvoxamine
10.	Growth hormone I.V or S.C.	10.	Gabapentin
11.	Lamotrigine	11.	Gamma - hydroxybutyric acid
12.	Liraglutide	12.	Leuprolide
13.	Metformin	13.	Lithium
14.	Miglitol	14.	Metoprolol
15.	Nefazodone	15.	Mirtazapine
16.	Octreotide	16.	Nateglinide
17.	Orlistat	17.	Nortriptyline
18.	Phentermine resin diethylpropion	18.	Olanzapine
19.	Pramlintide	19.	Paroxetine
20.	Sertraline^b^	20.	Pioglitazone
21.	Sitagliptin	21.	Propranolol
22.	Testosterone	22.	Quetiapine
23.	Topiramate	23.	Repaglinide
24.	Topiramate + Phentermine	24.	Risperidone
25.	Ziprasidone	25.	Terazosin
26.	Zonisamide	26.	Valproate
		27.	Venlafaxine

^a^All of these drugs do not fall into the listed classes.

^b^These drugs show a bimodal behavior, may cause weight loss in the first year and weight gain in the second year of use.

The ES commissioned this SR to inform its development of clinical practice guidelines for the management of obesity.

### Search for systematic reviews

The first author (JPD) will search MEDLINE, DARE, and the Cochrane Database of Systematic Reviews until at least two systematic reviews per drug are found. An expert reference librarian (LP) will provide assistance when needed.

### Eligible systematic reviews

We will search for SRs of RCTs that compare any drugs from our predefined list (Table 1) in enrolled adult patients.

We will include at least, if available, two SRs per drug to allow testing the effect of the drug on weight change in more than one setting. For instance, beta blockers are used in hypertension and in hyperthyroidism. Their effect on weight change will be different between the two conditions and will be tested in subgroup analysis.

If multiple SRs evaluated the same drug, we would choose the one with the most recent search date. If more than two shared the same search date or almost the same (<1 year apart), we would pick the one with the largest number of RCTs and with the most relevant clinical scenario for those drugs. For instance, we know that sertraline could be used in eating disorders but its main indication is major depression and obsessive-compulsive disorder. If there is no clear difference in the frequency of the use of the drugs by a specific condition, we will include the SRs without considering this criterion (for example, beta blockers for myocardial infarction or for essential hypertension). If we find more than two SRs with no clear rationale to select one over the other, we will include all of them.

### Search for individual studies

With input from study investigators with expertise in conducting SRs (MHM, VMM), a reference librarian (LP) and the first author (JPD) will design and execute electronic search strategies for the drugs that do not have any published relevant SRs. We will search electronic databases to identify relevant RCTs (Ovid Medline, OVID EMBASE, OVID Cochrane Library, Scopus, and PsycInfo) from their inception through September 2012.

Weight change is particularly subject to patients’ characteristics at baseline (confounders) and co-interventions that can occur during the course of a study (diet, exercise, psychological factors). Therefore, we will use RCTs to evaluate this outcome despite their known limitations compared to observational studies (smaller sample size, shorter follow-up and challenges of applicability due to the selectivity of patients).

### Eligibility criteria for the RCTs

We will include parallel or cross over RCTs that enrolled adults (>18 years old) and evaluate any drug listed in Table 1 (we will only include the listed drugs) as an intervention with a length of interventions no shorter than 7 days. Studies that investigated combinations of drugs (except for the listed ones) will be excluded; as well, we will exclude studies with inadequate outcome measurement (self-reported weight change). Considering the large number of included interventions (drugs) and for feasibility purposes, we only included randomized trials in which the control group received placebo and did not to include observational, quasi randomized, or trials in which the comparison was not placebo (another drug).

### Study selection

Agreement will be measured using the kappa or phi statistics, as appropriate (the latter is appropriate when the distribution of agreement is extreme). We are expecting over 5,000 references to screen and are prepared to have eight reviewers perform study selection. A reference management system (DistillerSR, Canada) will be used for study selection and provide real-time agreement statistics. The first author (JPD) will monitor the agreement during the RCT selection. He will call to meeting as needed in order to discuss disagreement and clarify the protocol and selection criteria.

Each abstract and title that result from executing the search strategy will be reviewed by at least two reviewers, in order to evaluate the potential eligibility of each of them.

Reviewers will request the full text versions of all potentially eligible studies. References associated with disagreements during abstract and title screening will also be also retrieved in full text for evaluation.

Two reviewers working separately and independently will consider the full text reports (all available versions of each study) for eligibility. The reviewers will calibrate their judgments using a smaller set of reports. Subsequently, disagreements will be resolved by consensus; if not possible, by arbitration.

### Data collection and extraction

Data extraction will include full descriptions of participants enrolled, the interventions they received (dose, frequency, route), the monitoring for efficacy or adherence, and the measure of outcome (specifically defined as event or measure and time frame for the ascertainment of this outcome). For studies with more than one follow-up period, we will select the longest.

### Risk of bias assessment

We will assess the methodological quality of RCTs using the Cochrane risk of bias tool to determine: how the randomization sequence was generated; how allocation was concealed, whether there were important imbalances at baseline; which groups were blinded (patients, caregivers, data collectors, outcome assessors, data analysts); what was the loss to follow-up; whether the analyses was by intention to treat; and how was missing outcome data dealt with. We will also analyze the adequacy of the outcome measurement process; for instance, the confidence will be higher in the RCTs that evaluated weight changes using a specific predefined protocol, for instance, three different measurements, all of those before breakfast using the same scale rather than RCTs that evaluate weight changes using one random measurement during the day. No scoring system will be derived for risk of bias assessment because calculating a summary score inevitably involves assigning ‘weights’ to different items in the scale, and it is difficult to justify the weights assigned and scales are less likely to be transparent to users of the review. We will present a table summarizing the risk of bias assessment, showing how each trial was rated on each criterion.

### Statistical analysis

Our primary outcomes will be: (1) weight loss and gain assessed as a continuous outcome (change in mean weight in kg or change in mean body mass index (BMI) in kg/m^2^); (2) dichotomous outcomes (defined as a number of patients with increased or decreased weight *vs*. total number of patients in each group. The primary continuous outcomes will be expressed as a mean difference (MD) in kg or kg/m^2^. We will calculate the MD considering the mean difference in weight or BMI change between baseline and the last available follow-up in both study arms (drugs and placebo). When needed, we will combine our primary continuous outcomes (for example, BMI changes from studies 1, 2, and 3, and weight changes from studies 4, 5, and 6) pooling the percentage of change of both. Our primary dichotomous outcome will be presented as a relative risk, comparing the ratio of the incidence of weight change in each trial arm.

**Figure 1:**
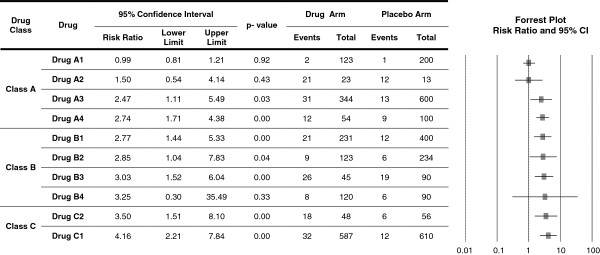
The risk of weight change (gain/loss).

**Figure 2:**
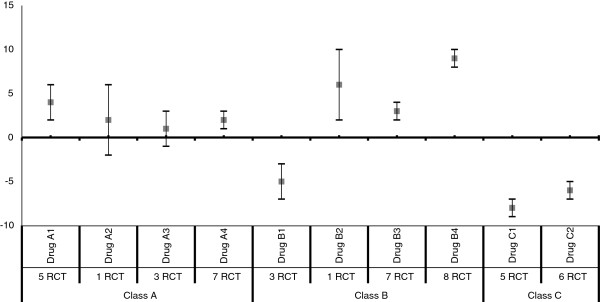
Mean difference and 95% confidence intervals for weight change in kilograms*.

As a secondary outcome, we will use the definition by Stevens *et al*. [7], a change of 5% or more (for example, 7% to 10%), either for weight gain or loss, of the baseline weight as a clinically important weight change, this is a more inclusive definition considering that most trials use cutoffs of 7% to 10% [8-10]. It will be presented as a relative risk. We prefer relative risk over odds ratio because it is more intuitive for clinicians [11]. We will present 95% confidence interval to distinguish between significant and non-significant drug effect on weight. Results will be presented graphically by drug and class in a forest plot as shown in Figures 1 and 2.

If enough data is available, we will adjust our primary outcomes according to their change rate over time, also we will categorize the included studies as <3 months, 3 to 6 months, and >6 months of interventions and they will be analyzed in their respective groups.

Our outcomes (primary and secondary) will be analyzed by drug and condition (for example, beta blockers in heart failure, hyperthyroidism, and hypertension). If the RCTs evaluated different doses of the same drugs first we will pool the effects within a study and also we will evaluate the differences on the weight change between each dose.

We will extract and evaluate outcomes using the intention-to-treat analysis. For studies with loss to follow-up, we will use the number of patients randomized as a denominator for the risk estimate. The decision aims at preserving randomization benefits in balancing prognosis of trial arms even if it led to underestimating the effect size [12].

We will conduct random-effects meta-analysis using the DerSimonian & Laird method to pool treatment effects from included studies [13].

We will use the I^2^ statistic and its 95% confidence interval [14] and Cochran’s Q test to assess heterogeneity across studies. Taking into account that thresholds for the interpretation of I^2^ can be misleading we will analyze its importance based on: (1) magnitude and direction of effects; and (2) strength of evidence for heterogeneity [15] (for example, *P* value from the Cochran’s Q test and confidence intervals for I^2^).We will explore heterogeneity by performing predefined subgroups analyses. Finally, publication bias will be assessed, whenever possible (sufficient number of studies, low heterogeneity), using the Egger regression asymmetry test, the Begg adjusted rank correlation test, and visual examination of funnel plots [16,17]. Analysis will be conducted using STATA version 12.0 (StataCorp, College Station, TX, USA).

### Dealing with missing data

To reduce the risk of selective outcome reporting; which is particularly problematic in studies evaluating harms or side effects (such as weight change) [18,19], we will attempt to contact by e-mail authors of RCTs that either did not report weight changes or did not report sufficient data for their inclusion in the meta-analysis (for example, standard deviation), we will do the same for RCTs that did not report sufficient details to evaluate the risk of bias. We will use a maximum of two contact attempts at 2-week intervals. After this period studies with enough data will be included in the meta-analysis.

Based on our experience and similar systematic reviews published before [20], we assume that some eligible RCTs will not report all relevant data needed for analysis. Commonly the missing data are the standard deviations (or other variability measures) which remain missing even after contacting the authors. In order to include these studies in the analysis, we will try these three steps in order: (1) calculate needed data elements from other reported statistics such as confidence intervals, *P*, or t values [15]; (2) impute the standard deviation from one large study of similar population and intervention [21]; (3) if no one large study is available to provide a reliable estimate of variability, we will use the mean of standard deviations across the studies in the same analysis. Any imputations or assumptions made in this step will be tested in a sensitivity analysis to ascertain robustness of conclusions.

### Subgroup and sensitivity analysis

We will conduct subgroup analyses per drug if sufficient data were available based on: (1) baseline weight category: obese (BMI ≥30) *vs*. non-obese (BMI <30); (2) gender (male *vs*. female); and (c) risk of bias of the included studies (low and unclear risk of bias *vs*. high risk of bias),

In order to assess the robustness of our result to missing imputed data the following sensitivity analyses will be conduct on the primary and secondary outcomes: exclusion of trials with imputed data. We will also conduct a sensitivity analysis to test if the effect size (weight change) is affected by: (1) drug daily dose, we will analyze range of doses between studies; (2) concomitant condition, we will analyze each condition by separately (for example, beta-blockers in heart failure will be analyzed separately from beta-blockers in hyperthyroidism).

The study will be reported in accordance with the recommendations set forth by the Preferred Reporting Items for Systematic Reviews and Meta-Analyses (PRISMA) work groups [22].

### Evaluating the quality of evidence

We will use the GRADE framework (grading of recommendations, assessment, development and evaluation) [23] to rate the quality of evidence supporting the weight change effect associated with each drug. This rating will reflect our confidence in the pooled estimate and will include the factors of methodological limitations of the studies, imprecision, indirectness, inconsistency, and reporting and publication biases. The quality will be rated as high, moderate, low, or very low.

## Discussion

This systematic review is based on an umbrella approach and aims to synthesize the available evidence about weight change associated with commonly prescribed drugs. This meta-analysis will offer the opportunity to generate a ranking and provide useful inferences within each class of obesogenic and leptogenic drugs based on its effect on weight, allowing guideline developers and patient-physician dyad to choose between available therapies.

### Limitations and strengths of this study

We will not conduct a primary search (SR *de novo*) for every drug because of the large number of included drugs (Table 1). Feasibility and the need for a comprehensive list led to this decision.

The list of drugs was selected by experts based on their knowledge of the field. They determined where guidance is needed for clinicians and patients; therefore, the choice of drugs is arbitrary.

This project represents a colossal effort and we agree that it is a unique adequate balance between rigor and feasibility that will provide the best evidence available to guideline developers, clinicians and patients to choose between available therapies based on their values and clinical context.

## Systematic review status

The systematic review is currently searching for eligible SRs. We expect to start the abstract screening of RCTs no further than the end of May 2012.

## Abbreviations

BMI: Body mass index; MD: Mean difference; RCTs: Randomized controlled trials; SRs: Systematic reviews.

## Competing interest

The authors declare that they have no competing interests.

## Authors’ contributions

All listed authors contributed substantially to the design of this protocol. All authors read and approved the final manuscript.

## *Funding*

This study was supported by the Endocrine Society by contract.
